# Use of Geosocial Networking Apps and HIV Risk Behavior Among Men Who Have Sex With Men: Case-Crossover Study

**DOI:** 10.2196/17173

**Published:** 2021-01-15

**Authors:** Justin Knox, Yi-No Chen, Qinying He, Guowu Liu, Jeb Jones, Xiaodong Wang, Patrick Sullivan, Aaron Siegler

**Affiliations:** 1 Columbia University New York, NY United States; 2 Emory University Atlanta, GA United States; 3 Center for Disease Control and Prevention Chengdu China; 4 Center for Disease Control and Prevention Beijing China; 5 Chengdu Tongle Social Work Service Center Chengdu China

**Keywords:** HIV, case-crossover study, dating apps, geosocial networking apps, men who have sex with men, sexual risk behavior

## Abstract

**Background:**

HIV disproportionately affects men who have sex with men (MSM) in China. The HIV epidemic is largely driven by unprotected anal sex (ie, sex not protected by condoms or HIV pre-exposure prophylaxis [PrEP]). The possible association between unprotected anal sex and the use of geospatial networking apps has been the subject of scientific debate.

**Objective:**

This study assessed whether users of a gay geospatial networking app in China were more likely to use condoms when they met their partners online versus offline. A case-crossover analysis, with each person serving as his own control, was employed to address the potential bias that men looking for sex partners through an online dating medium might have inherently different (and riskier) patterns of sexual behavior than men who do not use online dating media.

**Methods:**

A cross-sectional survey was administered in 2018 to adult male users of Blued—a gay geospatial networking app—in Beijing, Tianjin, Sichuan, and Yunnan, China. A case-crossover analysis was conducted among 1311 MSM not taking PrEP who reported engaging in both unprotected and protected anal sex in the previous 6 months. Multivariable conditional logistic regression was used to quantify the association between where the partnership was initiated (offline or online) and the act of unprotected anal sex, controlling for other interval-level covariates. Four sensitivity analyses were conducted to assess other potential sources of bias.

**Results:**

We identified 1311 matched instances where a person reported having both an unprotected anal sex act and a protected anal sex act in the previous 6 months. Of the most recent unprotected anal sex acts, 22.3% (292/1311), were initiated offline. Of the most recent protected anal sex acts, 16.3% (214/1311), were initiated offline. In multivariable analyses, initiating a partnership offline was positively associated with unprotected anal sex (odds ratio 2.66, 95% CI 1.84 to 3.85, *P*<.001) compared with initiating a partnership online. These results were robust to each of the different sensitivity analyses we conducted.

**Conclusions:**

Among Blued users in 4 Chinese cities, men were less likely to have unprotected anal sex in partnerships that they initiated online compared with those that they initiated offline. The relationship was strong, with over 2.5 times the likelihood of engaging in unprotected anal sex in partnerships initiated offline compared with those initiated online. These findings suggest that geospatial networking apps are a proxy for, and not a cause of, high-risk behaviors for HIV infection; these platforms should be viewed as a useful venue to identify individuals at risk for HIV transmission to allow for targeted service provision.

## Introduction

HIV disproportionately affects men who have sex with men (MSM) in China [1]. In 2014, a national meta-analysis reported a pooled 7% prevalence of HIV among MSM in China [2]. In Beijing, a cohort study [3] of MSM found an annualized HIV incidence of 5.9 per 100 person-years—an alarming level, similar to intense HIV epidemics among MSM in Thailand [4], South Africa [5], and the southern United States [6]. According to data from China’s national sentinel surveillance system, the HIV prevalence among MSM increased from 6% to 8% from 2010 to 2014 [7]. MSM in China represented 12% of new case diagnoses in the country in 2010 and 26% of new case diagnoses in 2014 [8]. MSM is the only risk group with increasing HIV diagnoses, and the estimated HIV incidence was higher for MSM in China than for any other key population [7]. The heightened risk of HIV infection among MSM is largely driven by unprotected anal sex (ie, intercourse not protected by condoms or HIV pre-exposure prophylaxis [PrEP]). Unprotected anal sex is one of the most efficient modes of HIV transmission, yielding a 17-times higher per-act transmission probability than vaginal sex [9,10].

An association between meeting partners through geospatial networking apps (eg, dating apps or “hook-up” apps) and having unprotected anal sex has been hypothesized [11-19]. As smartphone ownership grows increasingly ubiquitous worldwide [20], including in China where there are currently over 800 million smartphone users (more than one-half of the population) [21], there is concern that, if true and causal, such an association might contribute to increased levels of HIV transmission. Studies that have sought to explore this have produced mixed results: some studies conducted among MSM linked the use of geospatial networking apps to high-risk sexual behavior and adverse sexual health outcomes [11-17], but others found no association [18,19]. Mixed results have also been reported in China specifically, where one study reported a higher prevalence of HIV in MSM who met sex partners through the internet [17], while another found no difference in the number of condomless anal sex partners who were male between MSM who used partner-seeking mobile apps and those who did not [19].

**Figure 1:**
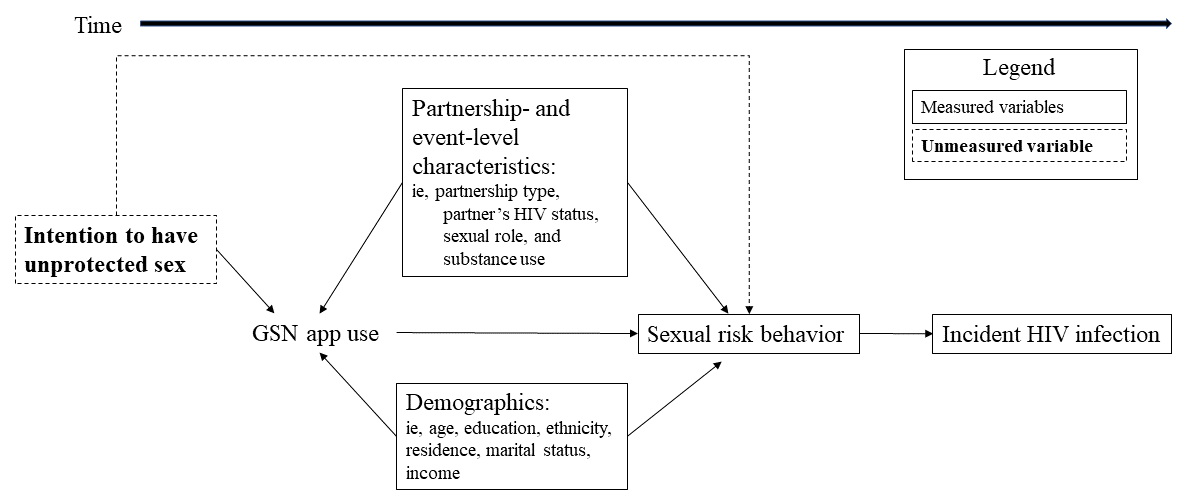
Directed acyclic graph showing the hypothesized pathway from geosocial networking (GSN) app use to sexual risk behavior and incident HIV infection, highlighting the unmeasured confounding effect of intention to have unprotected sex.

More recently, a study in China investigated the impact of the use of a geospatial networking app on incident HIV infection using longitudinal data from a cohort of MSM, finding that incident infection in the follow-up period was associated with ever using a dating app (but, interestingly, not with recent app use) [22]. A shortcoming of the study, and the others mentioned previously that examined the association between app use and HIV infection, is that a substantial source of confounding—the intention to have unprotected sex—was not adequately accounted for. As we elaborate using a directed acyclic graph in Figure 1, if one hypothesizes that exposure to a geospatial networking app causes an increase in HIV risk behaviors, which in turn causes increased HIV incidence, then intention to have unprotected sex (in dashed boxes with bold text) is a confounder because it is associated with both the exposure of interest (one reason that a person might download and use a geospatial networking app is their intention to have unprotected sex) and the outcome of interest (intention to have unprotected sex increases HIV risk, which in turn causes increased HIV incidence). Therefore, not accounting for this variable adequately would result in the observation of a spurious association, even in the event that the underlying variables of interest (ie, use of geospatial networking apps and HIV incidence) were not associated. Without taking intention to have unprotected sex into account, there is no way to determine whether using a dating app increases risk or (alternative hypothesis) people are using the dating app because they intended to have unprotected sex before they even downloaded it. To evaluate a potential causal association, a study design was needed to address the inherent selection bias that imbalances a comparison between men who choose to use dating apps and those who do not. The case-crossover design is one mechanism to address this potential bias in that each person serves as his own control, and the comparison is between the types of sexual activity during periods of exposure (with a partner met online, through a dating app or website) and the types of sexual activity in nonexposure periods (with a partner met offline).

Therefore, we used a case-crossover approach in order to assess whether users of a geospatial networking app in China were more likely to have sex not protected by condoms when they initiated partnerships online versus when they initiated partnerships offline. We used data from a cross-sectional study [23] conducted among users of Blued, the largest gay geospatial networking app in the world, with over 40 million registered users in China [24].

## Methods

### Recruitment

A cross-sectional survey was administered from May 6 to 17, 2018, to adult (≥18 years of age) Blued app users who were male at birth and located in Beijing, Tianjin, Sichuan, or Yunnan, China. Recruitment was conducted through the built-in advertising functions within the Blued app, which included pop-up messages, clickable links, banner advertising, and text message notifications. Text message notifications were sent to 631,963 randomly selected Blued users. Advertisements were designed by the study team and implemented by collaborators at Blued. A total of 34,701 Blued users clicked the link to the survey webpage. Advertisements asked potential participants if they would like to participate in a short research survey. Participants who clicked on the advertisements were taken to a survey platform designed by Sojump within the Blued app, which provided information about the study and a way to navigate to the eligibility screener. All eligible participants were consented electronically before completing the online survey, which was administered using the Sojump survey platform. Although 6040 Blued users initiated the survey process, 1368 of them did not complete the informed consent or did not meet the screening criteria, leaving 4672 participants eligible for these analyses. For analysis, data were exported from Sojump and stored on an encrypted computer at Blued in Beijing.

Ethical approval was provided by China’s National Center for AIDS/STD Control and Prevention (NCAIDS) (KX180117492), which is registered with the US Office for Human Research Protections (IRB0000227) and has a Federal Wide Assurance (FWA00002958).

### Measures

In the survey, participants were asked to describe their last sex acts involving anal sex with condom use and without condom use, respectively. This allowed us to create a “case interval” (ie, last act of unprotected anal sex) and a “control interval” (ie, last act of protected anal sex) for each individual participant. The survey also collected other characteristics of the sex act (ie, interval-level characteristics), including where participants initiated the partnership (online [eg, through Blued or another app or website] or offline [eg, through friends, at a bar, in a park]), the partnership type (ie, main or committed partner, casual partner with multiple sex acts, or casual partner with a single sex act [one-time]), the participant’s knowledge of the partner’s HIV status (ie, negative, positive, or unknown status), the participant’s role in anal sex (ie, receptive, insertive, or both), and the participant’s engagement in substance use (eg, alcohol or illicit drugs) before sexual intercourse.

The survey also collected data regarding the participant’s demographic characteristics, sexual identity, substance use in the past 6 months (rush poppers, methamphetamine, methylenedioxymethamphetamine, gamma-hydroxybutyrate, ketamine, and ecstasy), sexually transmitted infections (STIs) in the past 6 months, and HIV status.

### Statistical Analysis

#### Primary Analyses

The objective of the study was to test the association between the use of an online dating medium for finding sex partners and engagement in unprotected anal sex (without the use of condoms or PrEP). We used a case-crossover analysis to isolate the effect of initiating partnerships online (versus offline) on engagement in unprotected anal sex by comparing instances where men initiated partnerships through an online dating medium with instances where they initiated partnerships through an offline medium. Survey respondents who reported always or never using condoms during anal sex in the past 6 months were excluded because they were not informative with respect to the effect of meeting partners online or offline. In addition, respondents who were currently taking PrEP were excluded from the case-crossover analysis.

We used chi-square tests (or Fisher exact test if categorical data were sparse) and two-sample equal variance *t* tests (or Mann-Whitney *U* test if continuous data were not normally distributed) to compare the distribution of individual-level characteristics by participants’ eligibility status for the case-crossover analysis. We also compared the distribution of interval-level characteristics between participants who initiated partnerships online and those who initiated partnerships offline in both case and control intervals. We developed bivariable and multivariable models fitting conditional logistic regression to quantify the association between where the partnership was initiated (offline or online) and unprotected anal sex. In the multivariable model, partnership type, partner’s HIV status, participant’s role in anal sex, and participant’s substance use before sex were included as covariates. For all hypothesis tests conducted in this analysis, 95% CIs and a two-tailed *P* value of <.05 were used to assess statistical significance. All data were analyzed using SAS software (version 9.4; SAS Institute Inc).

#### Sensitivity Analyses

Sensitivity analyses were used to assess possible sources of bias in the analysis. First, the use of online dating media for finding sexual partners could in turn be a determinant of partnership type (ie, sex with one-time, casual, or main partner), which could be associated with unprotected anal sex. Thus, partnership type might be along the causal pathway between initiating a partnership offline and unprotected anal sex, making it a mediator of this relationship and therefore inappropriate to control for it as a potential confounder. To assess this possibility, we conducted multivariable analyses that did not include partnership type as a covariate (as it was in the main analyses). Second, to assess whether the association between initiating a partnership online and having unprotected anal sex varied by partnership type, we stratified the sample by partnership type and conducted analyses within strata. Third, to account for potential differences in sexual behavior among those who met partners exclusively offline or exclusively online, we restricted the analyses to participants who initiated partnerships both online and offline. Fourth, to account for potential differences in sexual behavior among HIV-positive men, we restricted the analyses to participants whose self-reported HIV status was either negative or unknown.

## Results

### Sample Characteristics

A total of 4672 participants completed the survey. Of those, 3361 participants (71.9%) were excluded from this analysis because they reported not having anal sex in the last 6 months (n=1215), never having condomless anal sex (n=267), always having condomless anal sex (n=1873), or currently using PrEP (n=6). This left a remaining sample of 1311 participants (28.1%) who reported data required for the case-crossover analysis: engaging in both protected and unprotected anal sex. Table 1 presents data on demographic characteristics, sexual orientation, drug use, HIV status, and STI status by inclusion in the analysis. The median age of the participants was 27 years (IQR 23-33 years). More than one-half (2751/4672, 58.9%) of the participants had completed college or more, 20.4% (952/4672) were students, and 71.9% (3360/4672) were employed. Most (3330/4672, 71.3%) of the participants identified as being homosexual. Compared with participants excluded from the analysis, participants included in the analysis were less likely to have attended college, were less likely to be students, were more likely to identify as homosexual, were more likely to report engaging in substance use in the past 6 months, were more likely to have had syphilis in the past 6 months, were more likely to have had gonorrhea in the past 6 months, and were more likely to be HIV positive.

**Table 1:** Characteristics of 4672 surveyed adult Blued app users in 4 provinces in China.

Characteristic			Total (N=4672), n (%)	Included in analysis (n=1311), n (%)	Excluded from analysis^a^ (n=3361), n (%)	*P* value
Age (years), median (IQR)^b^			27 (10)	27 (10)	27 (10)	.39
Ethnicity: Han Chinese			4332 (92.7)	1226 (93.5)	3106 (92.4)	.19
Education: college or above			2751 (58.9)	666 (50.8)	2085 (62.0)	<.001
**Current employment**						
	In the workforce		3360 (71.9)	935 (71.3)	2425 (72.2)	.57
	Student		952 (20.3)	234 (17.8)	718 (21.4)	.007
**Sexual orientation**						
	Homosexual		3330 (71.2)	993 (75.7)	2337 (69.5)	<.001
	Bisexual		1277 (27.3)	307 (23.4)	970 (28.9)	<.001
Substance use in past 6 months^c^			1611 (34.5)	661 (50.4)	950 (28.3)	<.001
**Self-reported STIs^d^ in past 6 months**						
	Not tested		3192 (68.3)	819 (62.5)	2375 (70.7)	N/A^e^
	Any STI		232 (5.0)	110 (8.4)	122 (3.6)	<.001
	Syphilis		126 (2.7)	65 (5.0)	61 (1.8)	<.001
	Gonorrhea		16 (0.3)	11 (0.8)	5 (0.1)	.003
	HPV^f^/genital warts		99 (2.1)	36 (2.7)	63 (1.9)	.50
**Self-reported HIV status**						
	Never tested		1416 (30.3)	295 (22.5)	1121 (33.4)	N/A
	HIV positive		406 (8.7)	160 (12.2)	246 (7.3)	<.001

^a^Participants who reported to never or always have had condom-protected anal sex 6 months prior to the survey collection were excluded from the study analysis because of the case-crossover study design.

^b^Age distribution was skewed. Wilcoxon Mann-Whitney *U* test was used to assess the distribution difference by eligibility. Median and IQR were used to characterize the distribution.

^c^Drugs included rush poppers, methamphetamine, methylenedioxymethamphetamine, gamma-hydroxybutyrate, ketamine, and ecstasy.

^d^STIs: sexually transmitted infections.

^e^N/A: Not applicable.

^f^HPV: human papillomavirus.

### Interval-Level Partnership Characteristics by Offline or Online Initiation, Stratified By Unprotected Anal Sex

Table 2 compares the distribution of interval-level characteristics in partnerships initiated offline and those initiated online within unprotected anal sex acts and within protected anal sex acts. Of the 1311 most recent unprotected anal sex acts, 1019 (77.7%) were initiated online. Of the 1311 most recent protected anal sex acts, 1097 (83.7%) were initiated online.

We found similar bivariate associations across case (unprotected sex) and control (protected sex) intervals. In both intervals, partnerships initiated offline were less likely to be with one-time partners (unprotected anal sex acts: 24.7% vs 52.6%, *P*<0.001; protected anal sex acts: 30.8% vs 54.9%, *P*<.001) and more likely to be with a partner who the participant believed to be HIV negative (unprotected anal sex acts: 42.1% vs 26.7%, *P*<.001; protected anal sex acts: 41.6% vs 23.2%, *P*<.001) than partnerships initiated online. In partnerships initiated offline, the participant was less likely to only engage in receptive anal sex (40.7% vs 49.3%, *P*=.02) and more likely to engage in substance use prior to having sex (10.7% vs 6.7%, *P*=.04) than in partnerships initiated online in the protected anal sex interval.

**Table 2:** Interval-level characteristics of sexual acts among 1311 adult Blued app users in 4 provinces in China who reported engaging in both unprotected and protected anal sex in the past 6 months.

Characteristic		All participants (N=1311)			UAI^a^				No UAI^b^		
		UAI^a^, n (%)	No UAI^b^, n (%)	Offline^c^ (n=292), n (%)		Online^d^ (n=1019), n (%)	*P* value	Offline^c^ (n=214), n (%)		Online^d^ (n=1097), n (%)	*P* value
**Partnership type**											
	One-time partner	608 (46.4)	668 (51.0)	72 (24.7)		536 (52.6)	<.001	66 (30.8)		602 (54.9)	<.001
	Casual partner	433 (33.0)	410 (31.3)	136 (46.6)		297 (29.1)	<.001	90 (42.1)		320 (29.2)	<.001
	Main partner	270 (20.6)	233 (17.8)	84 (28.8)		186 (18.3)	<.001	58 (27.1)		175 (16.0)	<.001
**Partner’s HIV status**											
	Negative	395 (30.1)	344 (26.2)	123 (42.1)		272 (26.7)	<.001	89 (41.6)		255 (23.2)	<.001
	Positive	45 (3.4)	55 (4.2)	12 (4.1)		33 (3.2)	.47	14 (6.5)		41 (3.7)	.06
	Not sure	871 (66.4)	912 (69.6)	157 (53.8)		714 (70.1)	<.001	111 (51.9)		801 (73.0)	<.001
**Participant’s sexual role**											
	Receptive	622 (47.4)	628 (47.9)	124 (42.5)		498 (48.9)	.05	87 (40.7)		541 (49.3)	.02
	Insertive	524 (40.0)	501 (38.2)	130 (44.5)		394 (38.7)	.07	92 (43.0)		409 (37.3)	.12
	Both	165 (12.6)	182 (13.9)	38 (13.0)		127 (12.5)	.80	35 (16.4)		147 (13.4)	.25
Participant’s substance use before sex (vs no use)		89 (6.8)	96 (7.3)	25 (8.6)		64 (6.3)	.17	23 (10.7)		73 (6.7)	.04

^a^UAI: unprotected anal sex interval.

^b^No UAI: protected anal sex interval.

^c^Offline: initiated partnership offline.

^d^Online: initiated partnership online.

### Associations Between Interval-Level Partnership Characteristics and Unprotected Anal Sex

Table 3 presents data on associations between interval-level covariates and unprotected anal sex. In multivariable analyses, the belief that partners were HIV negative (adjusted odds ratio [aOR] 1.57, 95% CI 1.09 to 2.27, *P*=.02) was associated with unprotected anal sex, compared with not being sure about the partner’s HIV status. Our primary outcome, initiating a partnership offline, was positively associated with unprotected anal sex (aOR 2.66, 95% CI 1.84 to 3.85, *P*<.001), compared with initiating a partnership online.

**Table 3:** Interval-level characteristics and unprotected anal sex among 1311 adult Blued app users in 4 provinces in China.

Characteristic		Bivariable model					Multivariable model^a^			
		OR^b^	95% CI	Wald χ^2^	*P* value	aOR^c^		95% CI	Wald χ^2^	*P* value
Partnership initiated offline (vs online)^d^		2.95	2.06-4.22	34.96	<.001	2.66		1.84-3.85	26.84	<.001
**Partnership type^e^**										
	One-time partner	Reference					Reference			
	Casual partner	1.41	1.08-1.83	6.51	.01	1.16		0.87-1.54	1.06	.30
	Main partner	1.68	1.24-2.27	11.13	.001	1.39		1.00-1.93	3.75	.05
**Partner’s HIV status^f^**										
	Negative	1.92	1.38-2.68	14.80	<.001	1.57		1.09-2.27	5.81	.02
	Positive	0.67	0.35-1.31	1.35	.25	0.58		0.29-1.15	2.45	.12
	Not sure	Reference					Reference			
**Participant’s sexual role^g^**										
	Receptive	Reference					Reference			
	Insertive	1.42	0.93-2.16	2.68	.10	1.47		0.95-2.28	3.01	.08
	Both	0.77	0.48-1.24	1.15	.28	0.74		0.45-1.19	1.55	.21
Participant’s substance use before sex (vs no use)^h^		0.72	0.39-1.32	1.13	.29	0.58		0.31-1.10	2.81	.09

^a^Model log-likelihood: –876.1.

^b^OR: odds ratio.

^c^aOR: adjusted odds ratio.

^d^Model log-likelihood: –888.6.

^e^Model log-likelihood: –902.0.

^f^Model log-likelihood: –899.6.

^g^Model log-likelihood: –905.6.

^h^Model log-likelihood: –908.1.

### Sensitivity Analyses

We conducted four sensitivity analyses in order to assess other potential biases. First, not including partnership type as a covariate in multivariable analyses had virtually no effect on the results (Multimedia Appendix 1). Second, the associations between initiating a partnership offline and unprotected anal sex stratified by partnership type were all positive and were not significantly different from each other (Multimedia Appendix 2). Third, restricting the sample to participants who met sexual partners both online and offline had virtually no effect on the results (Multimedia Appendix 3). Fourth, restricting the sample to participants whose self-reported HIV status was either negative or unknown had virtually no effect on the results (Multimedia Appendix 4).

## Discussion

### Principal Findings

In numerous previous assessments observing app users and comparing them with nonapp users, including several studies conducted in China, the causal model proposed was that exposure to a dating app caused an increase in HIV risk behaviors, which in turn caused increased HIV incidence [11-15,17,22]. We hypothesize that an underlying factor might make individuals who download, install, create an account, and use a dating app inherently different from those who do not: intention to have unprotected sex. In this concept, the app is a venue (akin to a bar or other meeting place), with intention to have unprotected sex causing both app use and subsequent unprotected sex with a higher HIV transmission risk. This analysis provides the first data to take into account the intention to have unprotected sex by those using dating apps by using a case-crossover design. Our primary finding—consistent across bivariable, multivariable, and sensitivity analyses—is that individuals were more likely to have unprotected anal sex in partnerships that they initiated offline compared with partnerships that they initiated online. The relationship was substantial, with over 2.5 times increased likelihood of engaging in unprotected anal sex in partnerships initiated offline compared with those initiated online.

Although our findings are in contrast to previous assessments that were mentioned, there are also studies that have reported similar results. For example, a study among young MSM using Grindr (a geosocial online dating app) found significantly higher rates of condom use with partners met on Grindr relative to partners that they met elsewhere [18]. However, these same authors reported in a subsequent study that familiarity with Grindr (using it for at least 1 year and meeting more partners through Grindr in the past month) was associated with increased sexual risk behavior (ie, condomless anal intercourse with their most recent Grindr-met partner) [16]. Specific to China, a study found that MSM who used partner-seeking mobile apps (eg, Jack’d, Grindr, Blued) did not report more condomless sex than men who did not use apps [19]. The current study improves upon these previous studies by looking within app users and employing a case-crossover design to avoid making potentially biased comparisons.

We also found that participants in a large majority of partnerships (68.0%) were not sure about their partner’s HIV status. There are two potential explanations for this. One explanation is that there is a high proportion of Chinese MSM who do not know their HIV status, which was reported in two previous studies, with 39.1% [25] and 33.2% [26] of MSM having never been tested for HIV. In this study, nearly one-third (30.3%) of the 4672 MSM from the entire sample had never been tested for HIV, and more than one-half (57.1%) had not been tested for HIV in the previous 6 months. Another potential explanation for the high frequency of sexual events among partners with unknown HIV status is that there is limited communication about and disclosure of HIV status among MSM prior to having sex, which has been reported previously [27]. Furthermore, partnerships were more likely to feature unprotected anal sex when a participant believed that his partner was HIV negative (compared with participants reporting that they were unsure of their partner’s HIV status). This type of safer sex negotiation in light of perceived risk has been reported previously in numerous studies [28,29].

### Limitations

We acknowledge several limitations in our study. First, data were cross-sectional and required recall from the previous 6 months (although we targeted a specific event in that time span, potentially reducing recall bias). Recall bias, if any, would be expected to bias toward the null. Second, data were based on self-report, and unprotected anal sex may have been under-reported. Again, this would be expected to bias toward the null. Third, although the comparisons made were within Blued app users and interval-level covariates were controlled for in multivariable analyses, there is the possibility that even though app use appears to be associated with condom use, this may not generalize to all sexual risk behaviors. For example, app users could have an elevated risk of HIV transmission through other means, such as by having more sexual partners.

### Conclusions

These limitations notwithstanding, ours is the first study to take into account the inherent intentions of individuals using geosocial networking apps, compared with those not using such apps, when looking at their effect on sexual risk behavior. Our assertion is that the factor motivating individuals to download, install, create an account, and use a dating app (ie, intent to have unprotected sex) contributes to the associations between app use and sexual risk behavior reported in previous studies. These findings have important policy implications. If dating apps are believed to contribute to increased risk of HIV transmission, then these apps might be perceived as a problem to be addressed by health agencies. However, if instead apps are a proxy for, and not a cause of, increased HIV risk, which our results suggest, then dating app platforms should be viewed as a useful venue to identify individuals at increased risk for HIV transmission to allow for targeted service provision such as HIV testing, condoms, and PrEP.

## Appendix

Multimedia Appendix 1 Interval-level characteristics (excluding partnership type) and unprotected anal sex among 1311 adult Blued app users in 4 provinces in China.

Multimedia Appendix 2 Associations between initiating a partnership offline and unprotected anal sex in three partnership-type strata among 1311 adult Blued app users in 4 provinces in China.

Multimedia Appendix 3 Interval-level characteristics and unprotected anal sex among 158 adult Blued app users in 4 provinces in China who initiated partnerships both offline and online.

Multimedia Appendix 4 Interval-level characteristics and unprotected anal sex among 1151 adult Blued app users in 4 provinces in China who did not self-report as HIV positive (HIV negative or unknown status).

## Abbreviations

aOR: adjusted odds ratio
MSM: men who have sex with men
NCAIDS: National Center for AIDS/STD Control and Prevention
OR: odds ratio
PrEP: pre-exposure prophylaxis
STI: sexually transmitted infection
