# Supplementary material for: Use of Geosocial Networking Apps and HIV Risk Behavior Among Men Who Have Sex With Men: Case-Crossover Study
Source: JMIR Public Health Surveill. 2021 Jan 15;7(1):e17173. doi: 10.2196/17173 (PMC7846440; doi:10.2196/17173)
Supplement: Multimedia Appendix 1 [file publichealth_v7i1e17173_app1.docx]

Multimedia Appendix 1. Interval-level characteristics (excluding partnership type) and unprotected anal sex among 1,311 adult Blue App users in 4 provinces in China.

|  | Multivariable model | | |
| --- | --- | --- | --- |
|  | aOR | 95% CI | *P* |
|  |  |  |  |
| **Characteristics** |  |  |  |
| **Partnership initiated offline (vs. online)** | 2.75 | 1.91 - 3.97 | <.001 |
| **Partnership type** |  |  |  |
| One-time partner | 1.75 | 1.24 - 2.48 | .002 |
| Casual partner | 0.60 | 0.31 - 1.19 | .15 |
| Main partner | Reference | | |
| **Participant sexual role** |  |  |  |
| Receptive | Reference | | |
| Insertive | 1.47 | 0.95 - 2.26 | .08 |
| Both | 0.75 | 0.47 - 1.22 | .25 |
| **Participant substance use before sex (vs. no use)** | 0.59 | 0.32 - 1.11 | .10 |
