# Supplementary material for: Use of Geosocial Networking Apps and HIV Risk Behavior Among Men Who Have Sex With Men: Case-Crossover Study
Source: JMIR Public Health Surveill. 2021 Jan 15;7(1):e17173. doi: 10.2196/17173 (PMC7846440; doi:10.2196/17173)
Supplement: Multimedia Appendix 2 [file publichealth_v7i1e17173_app2.docx]

Multimedia Appendix 2. Associations between initiating a partnership offline and unprotected anal sex in three partnership type strata among 1,311 adult Blue App users in 4 provinces in China.

|  | aOR | 95% CI | *P* |
| --- | --- | --- | --- |
|  |  |  |  |
| **Partnership type^a, b^** |  |  |  |
| One-time partnership (489 participants) | 1.27 | 0.64 – 2.49 | .49 |
| Casual partnership (287 participants) | 3.33 | 1.34 – 8.30 | .01 |
| Main partnership (150 participants) | 2.33 | 0.60 – 9.02 | .22 |

^a^ Wald test statistics comparing the stratum-specific odd ratios are as follows: 1) one-time v. casual: -1.760 (*P*=.08); 2) one-time v. main: -0.452 (*P*=.65); 3) casual v. main: 0.890 (*P*=.38)

^b^ Within each of the three partnership type strata we ran a multivariable model that including the main exposure (initiating a partnership offline) and any covariates associated with unprotected anal sex in bivariate analyses to obtain stratified adjusted odds ratios for initiating a partnership offline with unprotected anal sex
