# Supplementary material for: Use of Geosocial Networking Apps and HIV Risk Behavior Among Men Who Have Sex With Men: Case-Crossover Study
Source: JMIR Public Health Surveill. 2021 Jan 15;7(1):e17173. doi: 10.2196/17173 (PMC7846440; doi:10.2196/17173)
Supplement: Multimedia Appendix 3 [file publichealth_v7i1e17173_app3.docx]

Multimedia Appendix 3. Interval-level characteristics and unprotected anal sex among 158 adult Blue App users in 4 provinces in China who initiated partnerships both offline and online.

|  | Bivariable model | | | Multivariable model^a^ | | |
| --- | --- | --- | --- | --- | --- | --- |
|  | OR | 95% CI | *P* | aOR | 95% CI | *P* |
|  |  |  |  |  |  |  |
| **Characteristics** |  |  |  |  |  |  |
| **Partnership initiated offline (vs. online)** | 2.95 | 2.06 - 4.22 | <.001 | 2.70 | 1.83 - 3.98 | <.001 |
| **Partnership type** |  |  |  |  |  |  |
| One-time partner | Reference | | | Reference | | |
| Casual partner | 2.31 | 1.34 - 3.98 | .003 | 1.36 | 0.72 - 2.56 | .34 |
| Main partner | 2.50 | 1.2 - 5.21 | .01 | 1.55 | 0.67 - 3.57 | .31 |
| **Partner's HIV status** |  |  |  |  |  |  |
| Negative | 3.15 | 1.55 - 6.41 | .002 |  |  |  |
| Positive | 0.42 | 0.04 - 4.17 | .46 |  |  |  |
| Not sure | Reference | | |  | | |
| **Participant sexual role** |  |  |  |  |  |  |
| Receptive | Reference | | | Reference | | |
| Insertive | 1.38 | 0.46 - 4.18 | .57 | 0.89 | 0.25 - 3.19 | .86 |
| Both | 0.66 | 0.2 - 2.14 | .49 | 0.50 | 0.13 - 1.96 | .32 |
| **Participant substance use before sex (vs. no use)** | 1.25 | 0.34 - 4.66 | .74 |  |  |  |

^a^ Partner’s HIV status and participant substance use before sex were not adjusted in the multivariate model because of issues with multicollinearity.
