# Supplementary material for: Use of Geosocial Networking Apps and HIV Risk Behavior Among Men Who Have Sex With Men: Case-Crossover Study
Source: JMIR Public Health Surveill. 2021 Jan 15;7(1):e17173. doi: 10.2196/17173 (PMC7846440; doi:10.2196/17173)
Supplement: Multimedia Appendix 4 [file publichealth_v7i1e17173_app4.docx]

Multimedia Appendix 4. Interval-level characteristics and unprotected anal sex among 1,151 adult Blue App users in 4 provinces in China who did not self-report as HIV positive (HIV-negative or unknown status).

|  | Bivariable model | | | Multivariable model^a^ | | |
| --- | --- | --- | --- | --- | --- | --- |
|  | OR | 95% CI | *P* | aOR | 95% CI | *P* |
|  |  |  |  |  |  |  |
| **Characteristics** |  |  |  |  |  |  |
| **Partnership initiated offline (vs. online)** | 3.30 | 2.24 - 4.88 | <.001 | 3.03 | 2.03 - 4.52 | <.001 |
| **Partnership type** |  |  |  |  |  |  |
| One-time partner | Reference | | | Reference | | |
| Casual partner | 1.47 | 1.12 - 1.94 | .006 | 1.28 | 0.95 - 1.71 | .10 |
| Main partner | 1.85 | 1.34 - 2.55 | <.001 | 1.67 | 1.19 - 2.33 | .003 |
| **Partner's HIV status** |  |  |  |  |  |  |
| Negative | 2.09 | 1.48 - 2.95 | <.001 |  |  |  |
| Positive | 0.70 | 0.3 - 1.61 | 0.40 |  |  |  |
| Not sure | Reference | | |  | | |
| **Participant sexual role** |  |  |  |  |  |  |
| Receptive | Reference | | | Reference | | |
| Insertive | 1.40 | 0.91 - 2.17 | .13 | 1.36 | 0.87 - 2.12 | .18 |
| Both | 0.79 | 0.49 - 1.3 | .35 | 0.75 | 0.45 - 1.25 | .27 |
| **Participant substance use before sex (vs. no use)** | 0.74 | 0.4 - 1.38 | .35 | 0.6 | 0.31 - 1.15 | .12 |

^a^ Partner’s HIV status was not adjusted in the multivariate model because of issues with multicollinearity.
